# *Clumppling 2.0*: A Clustering Alignment Program for Population Structure Analyses

**DOI:** 10.47248/hpgg2606020004

**Published:** 2026-03-17

**Authors:** Xiran Liu, Noah A. Rosenberg, Sohini Ramachandran

**Affiliations:** 1. Data Science Institute, Brown University, Providence, RI, 02912, USA; 2. Department of Biology, Stanford University, Stanford, CA, 94305, USA; 3. Ecology, Evolution, and Organismal Biology, Brown University, Providence, RI, 02912, USA

**Keywords:** admixture, alignment, clustering, genetic ancestry, population structure

## Abstract

We previously introduced Clumppling to address the “alignment problem” for multiple mixed-membership unsupervised clustering results in population structure analyses, where clusters represent latent genetic ancestries. This problem stems from three challenges—label-switching, multi-modality, and varying numbers of clusters—which Clumppling resolves in three steps: aligning results with the same number of clusters, detecting distinct solutions or “modes,” and aligning modes across different numbers of clusters. Here, we present Clumppling 2.0, an update with features for visualizing the emergence of clusters, comparing aligned results from different models, and incorporating modularity of algorithmic steps. We outline the Clumppling 2.0 workflow, highlighting its improved algorithmic flexibility and visual interpretability through a graph of alignment patterns. We then demonstrate its utility on human genetic datasets that include individuals from admixed populations.

## 1. Introduction: The Clustering Alignment Problem in Population Structure Analysis

Population structure analysis uses clustering methods on genotype data to model individual genomes as mixtures of contributions from multiple sources or latent clusters. When prior information about cluster membership is unavailable, these clusters are inferred directly from genotype data using *unsupervised mixed-membership clustering* [1–3]. Methods for this inference include Structure [1,4], BAPS [5], Frappe [6], ADMIXTURE [7], and fastStructure [8], which have collectively been referenced tens of thousands of times. However, independent clustering runs on the same data can yield substantially different results due to stochasticity and the existence of multiple optima, complicating the interpretation of cluster membership patterns. A common practice is to perform multiple clustering runs, often with different random initializations, to avoid obtaining a single solution by chance and to identify reproducible patterns of population structure. Analyzing the output from multiple clustering runs introduces what is known as the “**cluster alignment problem**,” with three main challenges [9–13].

### Label-switching.

An unsupervised clustering method is equally likely to produce any of the *K*! permutations of the same set of cluster labels. These solutions are identical after appropriate permutation.

### Multi-modality.

**Figure 1.**
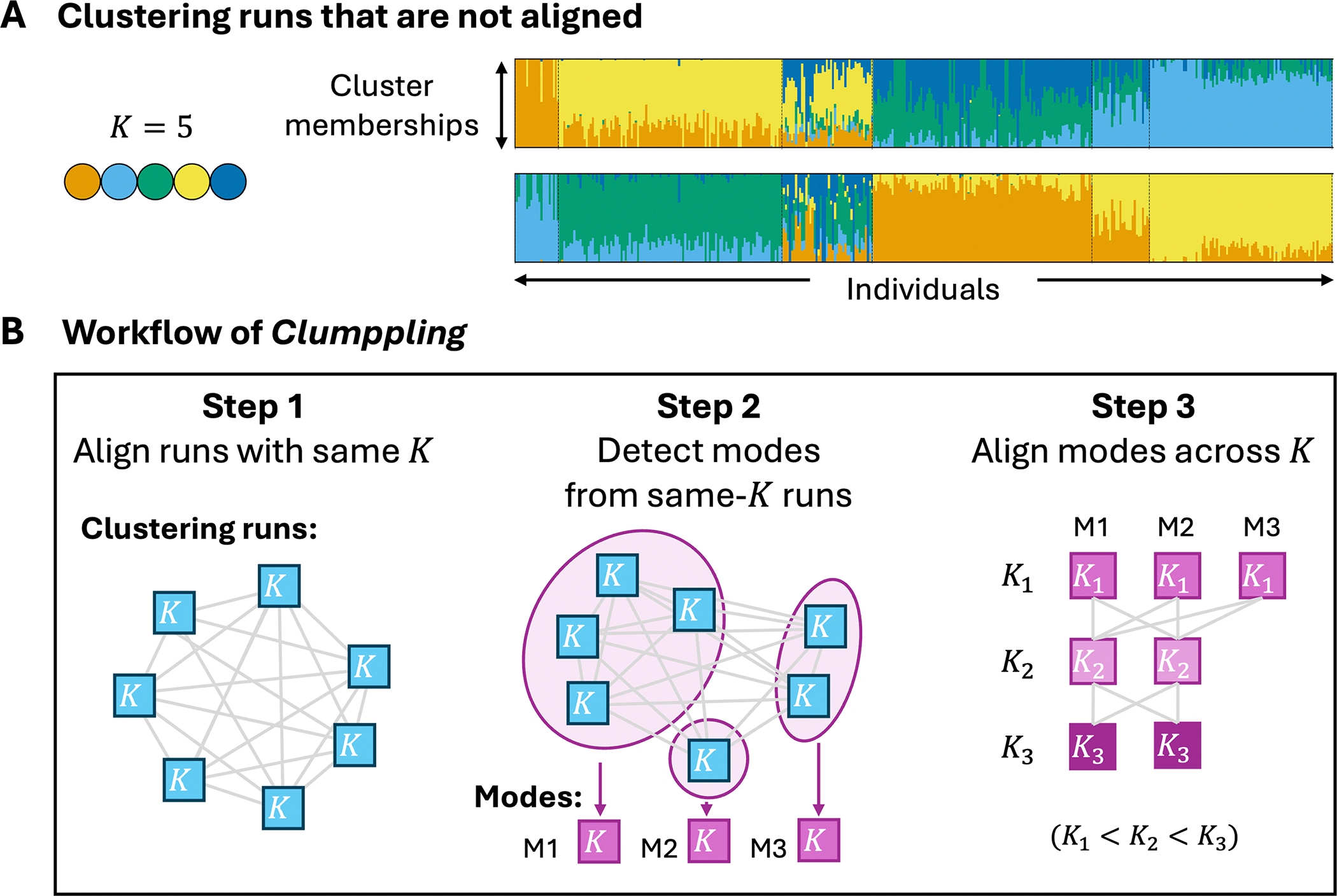
Unaligned clustering results and the workflow of Clumppling. (A) Bar plots from two example runs with *K* = 5 clusters, illustrating substantially different results. Unaligned plots are difficult to compare. (B) The workflow of Clumppling to perform clustering alignment, with three main steps. A “mode” is a clustering solution that is distinct up to the permutation of cluster labels.

Clustering runs can also produce truly distinct solutions (i.e., “modes”) that are not equivalent under any label permutation (e.g., Figure 1A). Multi-modality can arise from the convergence of the clustering algorithm to different local or global optima, all providing plausible explanations for the data.

### Different numbers of clusters (*K*) across runs.

In unsupervised clustering, the number of clusters (*K*) typically needs to be specified by the user or inferred by the algorithm. Researchers often explore a range of values for *K*, but selecting a single optimal value is challenging and risks overlooking aspects of the population structure that are salient only at other values of *K*. Quantitatively examining results of multiple *K* values simultaneously can also be challenging, motivating alignment of runs across different *K*.

Multiple approaches have been developed to address these clustering alignment challenges, including Clumpp [9], Clumpak [10], and Pong [11]. The most recent advancement, our Clumppling approach [13], uses integer linear programming (ILP) and network strategies to efficiently tackle the alignment problem. The initial version of Clumppling combined some of the features of earlier methods, including systematic detection of modes in similarity networks, use of algorithms based on past work in combinatorial optimization, and computational efficiency, and it also considered new scenarios, such as alignment between non-consecutive values of *K* and alignment of mode pairs between adjacent *K* values [13].

## 2. Application

### 2.1. Clumppling: workflow and visualization

Clumppling [13] aligns clustering runs in three steps (Figure 1B): aligning clustering runs with the same *K* value, detecting modes among these runs, and aligning modes across *K* values.

First, Clumppling aligns clustering runs with the same number of clusters (*K*). For each such pair of clustering runs, Clumppling formulates the one-to-one matching of clusters as an optimization problem and solves it using ILP. The optimal solution (i.e., the one with the highest similarity) gives the alignment between the two runs.

Next, Clumppling detects “modes” from aligned same-*K* runs. By constructing a network with *R* runs as the nodes and with edges weighted by optimal alignment similarities for all $\left(\frac{R}{2}\right)$ pairs of runs, Clumppling adopts community detection to identify modes. These modes represent distinct solutions from the *R* runs. At the extremes, community detection can generate *R* singleton modes if all runs differ substantially, or a single mode of size *R* if all differences across runs are attributable to label-switching.

Finally, Clumppling aligns clustering modes across different values of *K*. It uses ILP to align a pair of modes between two adjacent *K* values (e.g., *K*_1_ *<K*_2_). For each (*K*_1_, *K*_2_), it designates an “anchor mode pair”—according to the user’s choice—as either the pair of modes with the highest alignment similarity or the major modes of size *K*_1_ and *K*_2_, that is, those that are most prevalent across runs. This anchor pair then serves as the reference for aligning all other modes with number of clusters *K*, *K* = *K*_1_ or *K* = *K*_2_.

**Figure 2.**
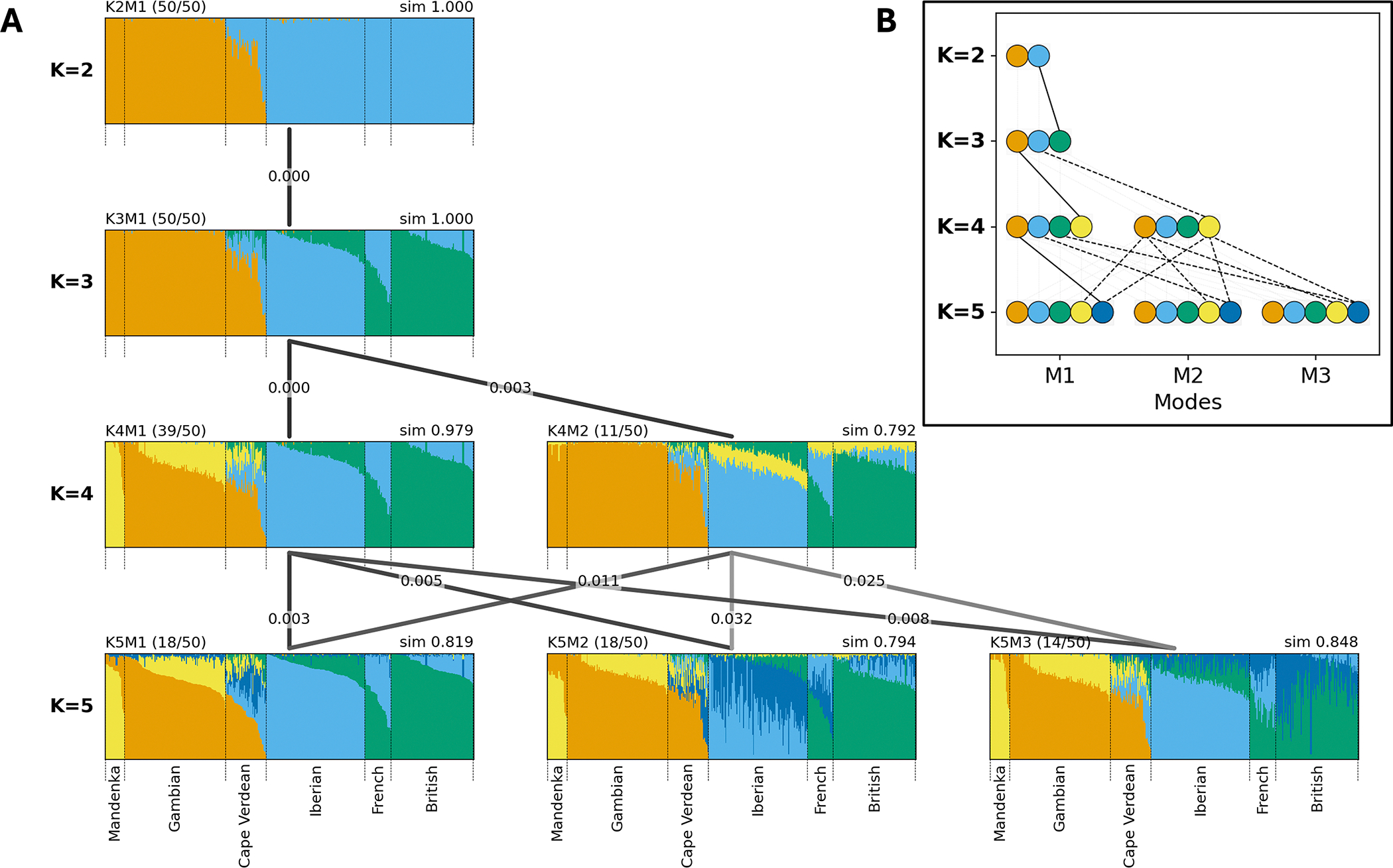
Clustering results from Cape Verde data aligned by Clumppling. There are 50 clustering runs for each *K* from 2 to 5. Detected modes are labeled, for example, “K2M1” (mode 1 of *K* = 2). (A) Clumppling‘s multipartite graph of bar plots, showing the aligned memberships in each mode. For each bar plot, the mode size (i.e., the number of clustering runs contained in that mode) of all runs with the given *K* is shown in parentheses at the upper left, and the within-mode alignment similarity is shown at the upper right (e.g., sim=0.979). Modes with numbers of clusters *K* and *K* + 1 are connected by lines, where edge labels indicate alignment cost and edge color reflects alignment similarity. Darker edges indicate mode pairs with higher similarity, whereas lighter edges indicate poorer alignment. Individuals are grouped and labeled according to their population (provided as auxiliary labels by the user). Within each label group, individuals are displayed in decreasing order (left to right) by their membership in the cluster with the largest total membership in one specific plot (here *K*5*M*1); individuals are placed in this same order for all modes and all *K*. (B) The alignment pattern graph in Clumppling 2.0, showing the cluster to which each newly emerged cluster aligns. The layout and coloring of the modes match those of the multipartite graph in panel (A). Each node represents a cluster, with connecting lines indicating alignment between them. The newly-emerged nodes as *K* increases are appended to the right. To emphasize newly formed clusters, connections between directly-matched clusters (e.g., Cluster 1 to Cluster 1) are dimmed, and only connections for those not directly matched are shown clearly. Lines connecting clusters from an anchor pair are solid; all others are dashed.

The results of a single clustering run are often visualized as a bar plot, where each individual’s membership coefficients across the *K* clusters are displayed as stacked, color-coded bars (see Figure 1A for examples with *K* = 5). To display its aligned clustering results, Clumppling [13] arranges the bar plots of aligned modes from different values of *K* into a multipartite graph. Modes are arranged in rows by *K* value, and edges connect modes in adjacent rows. Edge color encodes alignment similarity, and edge labels report the optimal alignment cost (i.e., the objective value in Eq. 12 of [13]). Darker edges indicate higher similarity: the maximum-similarity pairs (e.g., in Figure 2A and Supplementary Figure S1A, pairs with similarity = 1 and alignment cost = 0) are colored black, whereas lower-similarity pairs are shown in progressively lighter shades of gray.

### 2.2. New features in Clumppling 2.0

Clumppling 2.0 introduces several updates, primarily focused on new visualizations, model comparisons, and algorithmic flexibility, including modularity of the steps of the analysis.

#### 2.2.1. Novel visualizations

Clumppling 2.0 introduces the ***alignment pattern graph*** as a key feature to trace cluster emergence and to reveal hierarchical population structure (see Figure 2B). Within this graph, modes are arranged and colored consistently with the multipartite graph view of bar plots (Figure 2A), but they are represented as a collection of *K* nodes, one per cluster. Clusters are connected between modes with adjacent *K* values (e.g., from *K* to *K* + 1) if they are aligned to one another. To highlight the emergence of new clusters, our visualization prominently displays connections to the newly formed cluster (e.g., the (*K* + 1)-th cluster) while dimming all other connections. For a non-anchor mode pair (indicated by dashed lines, in contrast to the solid lines for anchors), multiple connecting lines may exist (e.g., from *K*4*M*2 to *K*5*M*2 in Figure 2B). This new feature illustrates how clusters emerge and change across different values of *K*. By visualizing these sequential relationships, the alignment pattern graph provides an intuitive view of the hierarchical decomposition in population structure and offers insights into inferred memberships.

Clumppling 2.0 also includes an option to ***reorder individuals and clusters*** to improve the visual clarity of bar plots. When this option is used and auxiliary labels are provided by the user (e.g., population names), individuals are first grouped by their labels and then sorted within each labeled group in descending order of membership in that group’s dominant cluster (i.e., the cluster with the largest total membership). The sorting order is determined by cluster memberships from a user-selected reference—either the major mode with largest *K* or that with smallest *K*—and it is applied across all other modes (e.g., plots in Figure 1B are sorted based on *K*5*M*1). The reordering feature can be applied to clarify the cluster composition in each labeled group.

#### 2.2.2. Model comparison

**Figure 3.**
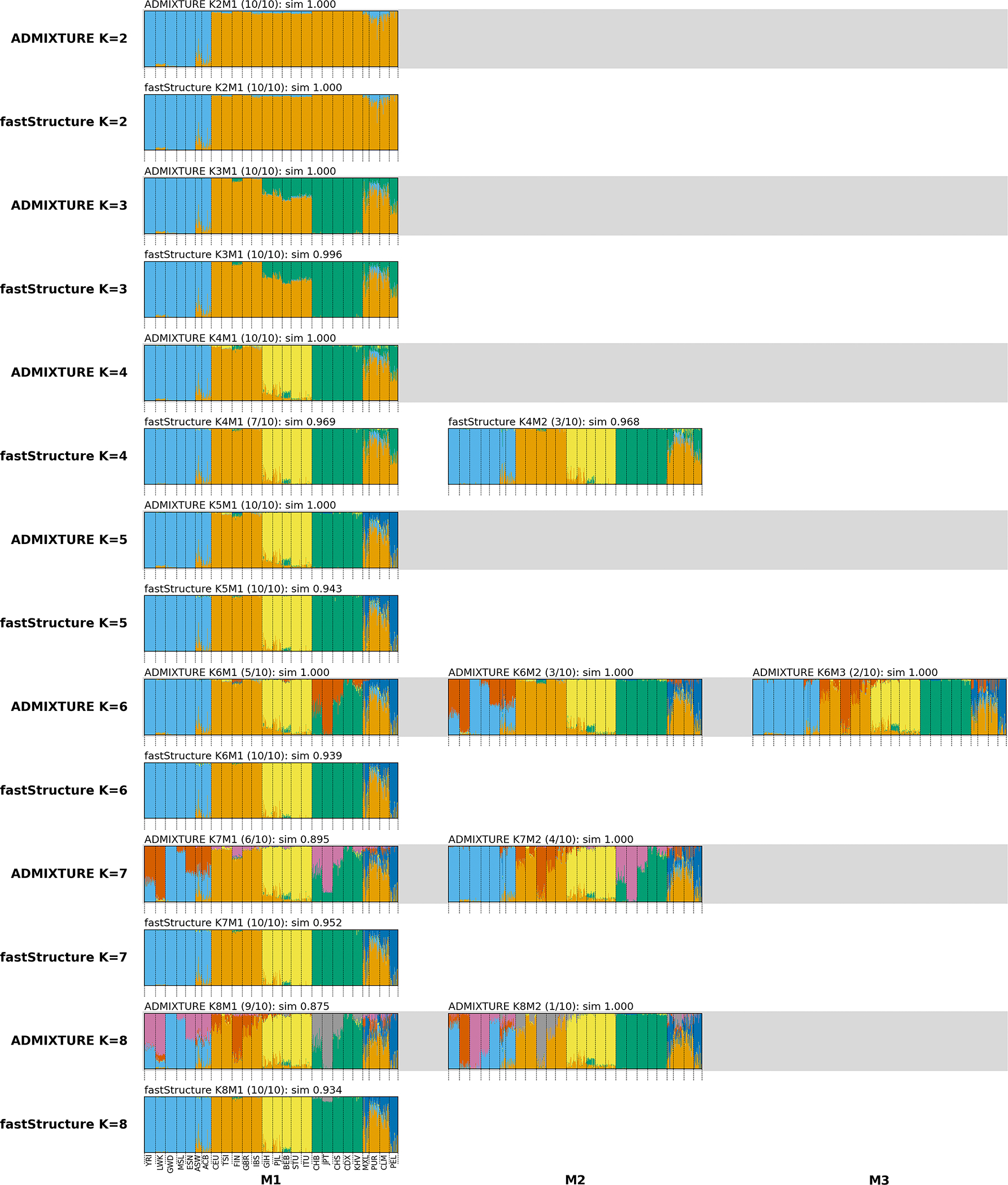
Model comparison of ADMIXTURE and fastStructure on data from the 1000 Genomes Project. Clumppling detects and aligns modes from each of the two methods’ clustering outcomes on the same data. The modes are then aligned across these two methods. The gray rows display the modes from ADMIXTURE, and the unshaded rows display the modes from fastStructure. This comparison highlights how the two methods approach population structure inference differently. See Supplementary Figure S1 for the full names of the 1000 Genomes Project populations.

Another new feature in Clumppling 2.0 is an interleaving-by-*K* ***model comparison*** feature, available as a submodule compModels in the program. Different “models” can refer to different *clustering methods* (e.g., Structure versus ADMIXTURE), *methodological settings* (e.g., the block relaxation versus the EM algorithm for ADMIXTURE optimization), or *downstream analytical choices* (e.g., Louvain [14] versus Markov clustering [15] algorithm for community detection in Step 2 of Clumppling). This feature is designed for comparing results from different models. Instead of summarizing all runs into a single set of modes globally, it first identifies representative modes for each model separately, then displays these per-model modes next to each other for direct comparison. The figure is designed with rows organized by model and grouped by *K*. The value of *K* increases from top to bottom, and rows for each model are distinguished by an alternating background color. An example is provided in Figure 3. On data from the 1000 Genomes Project [16], fastStructure [8], which explicitly encourages empty clusters, starts producing these clusters for *K* > 5, whereas ADMIXTURE [7] continues to yield interpretable clusters beyond *K* = 5. For example, in mode 1 of *K* = 6 (labeled as *K*6*M*1), ADMIXTURE resolves fine-scale differences among East Asians (CHS, CHB, JPT), illustrating the more refined subdivision compared to the structure detected by fastStructure and highlighting the importance of examining a range of *K* values. Model comparison also allows researchers to assess the robustness of inferred admixture patterns by checking for consistency across different analytical approaches.

#### 2.2.3. Improved algorithmic flexibility

Clumppling 2.0 features a ***modularized workflow***, where each step in Figure 1B can be run independently as a module or as part of the integrated pipeline. The modularity allows users to execute specific steps for targeted analyses or to substitute modules with custom approaches (provided the input and output formats are compatible), expanding the range of possible usage scenarios. It also provides greater user control over the analysis pipeline. For example, users can choose among multiple mode-detection algorithms and specify thresholds for defining modes (e.g., users can force all runs into a single mode if all pairwise alignment dissimilarities fall below a specified tolerance such as 10^−6^; this option is useful when minor disagreements are acceptable for summarization, even if hypothesis tests suggest nontrivial community structure). A full list of features is available in the software repository and user manual at https://github.com/PopGenClustering/Clumppling.

## 3. Implementation: Demonstrations on Human Genetic Datasets

The Python program Clumppling 2.0 requires the membership output of a set of clustering runs as the input, as provided by popular population structure inference methods (e.g., Structure, ADMIXTURE, fastStructure). It outputs alignment patterns within same-*K* runs and across modes of different *K*, along with alignment statistics, summary statistics of detected modes, aligned membership matrices, and visualizations of the aligned modes in a multipartite graph and an alignment pattern graph (see Figure 2). The run time of each ILP alignment is polynomial with respect to *K* (typically 2–40), making the method efficient in practice; bar-plot visualization (one bar per individual) may be slow for large cohorts. Excluding figure generation, Clumppling typically adds negligible computing time (a few seconds) relative to the clustering step.

### 3.1. Cape Verde data

We illustrate the new features of Clumppling 2.0 using clustering results from a genome-wide genotype dataset of 399 individuals, including 44 from Cape Verde and the remainder from African and European populations [17]. We applied Clumppling 2.0 to the runs reported by [17], aligning a total of 200 ADMIXTURE runs, with 50 runs for each *K* from 2 to 5. The aligned modes are shown in Figure 2A. The alignment pattern graph in Figure 2B presents how new clusters emerge. For instance, in the transition from *K* = 2 to *K* = 3, the new green cluster in mode *K*3*M*1 splits from the light blue cluster in mode *K*2*M*1, indicating fine-scale population structure within individuals of European ancestry.

The alignment pattern graph and bar plots (Figure 2) reveal that at *K* = 5, multiple plausible explanations exist for the clustering patterns, represented by three modes. The most common mode (*K*5*M*1; size=18, similarity=0.82) suggests a new, dominant cluster emerges mainly in Cape Verdean individuals (dark blue). However, an equally frequent mode with lower pairwise alignment similarity (*K*5*M*2; size=18, similarity=0.80) indicates a new cluster instead among Iberian and French individuals. A third well-supported mode (*K*5*M*3; size=14, similarity=0.85) points to a new cluster separating from the dominant cluster for French and British samples. The presence of these alternative modes indicates that the algorithm is concurrently resolving distinct, fine-scale structure within several groups, and that analyzing a single clustering run at a single value of *K* is insufficient for interpreting admixture patterns. When multiple modes are identified, we recommend prioritizing modes that are both frequently observed across independent runs (larger size) and internally consistent (higher within-mode alignment similarity). These criteria are complementary: larger mode size often reflects greater support, while within-mode similarity captures how coherently the runs in a mode agree with one another. In practice, we prioritize more frequently occurring modes, while flagging rare but highly consistent modes as potentially meaningful alternatives that may reflect distinct local optima or biologically relevant substructures.

### 3.2. 1000 Genomes Project data

We also performed a comparative analysis of ADMIXTURE [7] and fastStructure [8] using data from the 1000 Genomes Project [16], which includes 2,504 individuals from 26 labeled groups and 178,593 SNPs after pruning. For each of the two methods, we conducted 10 independent runs for *K* ranging from 2 to 8 on the pruned data using their default parameters, varying the random seed from run to run (see Appendix A for details of SNP pruning and other parameters). We applied clustering alignment to the membership matrices of each method separately before comparing their outcomes. See Supplementary Figure S1 for the aligned ADMIXTURE results and Figure 3 for the model comparison.

The results from the two methods are similar for *K* = 2 to 5, but they diverge at higher *K* values. fastStructure [8] is known to encourage formation of empty clusters, as shown in the clustering results beginning at *K* = 6, where the algorithm produces new clusters with negligible membership. However, ADMIXTURE still reveals nontrivial splits at larger *K* values. For example, the new red cluster in mode *K*6*M*1 corresponds to fine-scale population structure in East Asian groups (Figure 3 and Supplementary Figure S1). Modes *K*6*M*2 (African groups) and *K*6*M*3 (European groups) also correspond to meaningful substructure.

## 4. Discussion

We have introduced Clumppling 2.0, a framework for clustering alignment in population structure analysis. It provides several improvements over the initial release, with a focus on interpretability and analytical flexibility.

The novel visualization of alignment patterns helps clarify the hierarchical nature of population structure by tracking newly inferred clusters as the number of clusters increases. The new model comparison module allows researchers to evaluate the consistency of results across different analytical choices and helps guide the selection of appropriate data analysis methods. The framework also offers greater algorithmic flexibility and a modular design. These improvements contribute to a clustering alignment framework that can adapt to diverse datasets and methodological choices. The new features facilitate the examination of multi-modality, comparing solutions across *K* and across models, and communicating uncertainty and stability in population-structure inference.

## Supplementary Material

HPGG2606020004SupplementaryMaterials

The following supplementary materials are available on the website of this paper:

1. Appendix A. Population structure analysis of 1000 Genomes Project data.

2. Figure S1. Clustering results from the 1000 Genomes Project data aligned by Clumppling.

## Data Availability

All data used in this study are publicly available either through the cited references or via the program’s GitHub repository at https://github.com/PopGenClustering/Clumppling.
