# Supplementary material for: Clumppling 2.0: A Clustering Alignment Program for Population Structure Analyses
Source: Hum Popul Genet Genom. Author manuscript; Available in PMC 2026 May 13. (PMC13167144; doi:10.47248/hpgg2606020004)
Supplement: HPGG2606020004SupplementaryMaterials [file NIHMS2169305-supplement-HPGG2606020004SupplementaryMaterials.zip › HPGG2606020004SupplementaryMaterials.pdf]

Human Population Genetics and Genomics
https://doi.org/10.47248/hpgg2606020004

Technical Note

Clumppling 2.0: A Clustering Alignment Program for Population Structure Analyses

Xiran Liu [1,*], Noah A. Rosenberg [2], Sohini Ramachandran [1,3]

1. Data Science Institute, Brown University, Providence, RI, 02912, USA; Email: sramachandran@brown.edu
2. Department of Biology, Stanford University, Stanford, CA, 94305, USA; Email: noahr@stanford.edu
3. Ecology, Evolution, and Organismal Biology; Brown University, Providence, RI, 02912, USA

* **Correspondence:** Xiran Liu; Email: xiran_liu1@brown.edu

Supplementary Materials

The following supplementary materials are available on the website of this paper:

1. Appendix A. Population structure analysis of 1000 Genomes Project data.
2. Figure S1. Clustering results from the 1000 Genomes Project data aligned by `Clumppling`

1. Appendix A. Population structure analysis of 1000 Genomes Project data

There are 2,504 individuals from 26 labeled groups in the 1000 Genomes Project dataset [1]. The phase 3 data were downloaded from https://www.internationalgenome.org/data. After converting the VCF files to BED files, and pruning loci for linkage disequilibrium via `PLINK2` command "`--maf 0.05 --indep-pairwise 50 5 0.1`", as suggested in `ADMIXTURE`'s manual, 178,593 SNPs remain.

For `ADMIXTURE`, we used the block relaxation algorithm for optimization ("`--method=block`"), the default termination criterion that stops when the log-likelihood increases by less than 10^{-4} between iterations ("`--C=10e-4`"), and the quasi-Newton convergence acceleration method with $q = 3$ secant conditions ("`--acceleration=`qn3").

For `fastStructure`, we used the default convergence criterion of 10^{-6} ("`--tol=10e-6`"), the simple Beta(1,1) prior on allele frequencies ("`--prior=simple`"), and no cross-validation ("`--cv=0`").

We varied the random seed from run to run by setting "-s" in `ADMIXTURE` and "`--seed`" in `fastStructure`.

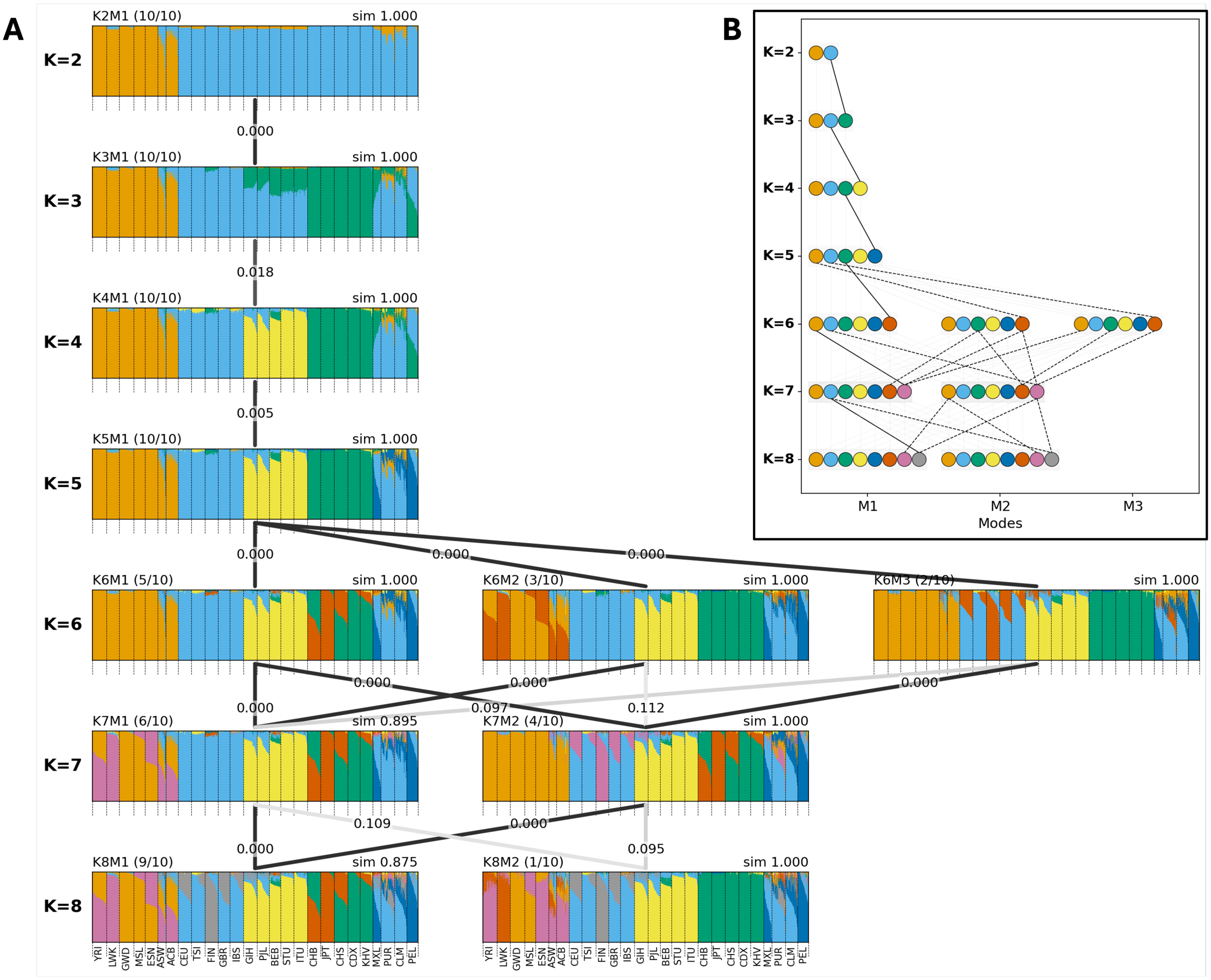

Figure S1. Clustering results from the 1000 Genomes Project data aligned by `Clumppling`. Clustering results include 10 `ADMIXTURE` clustering runs for each K from 2 to 8, generated on 2,504 individuals from 26 groups from the 1000 Genome Project data [1]. The layout follows that of Figure 2. (A) The multipartite graph of bar plots showing the aligned memberships in each mode. (B) The alignment pattern graph, showing the cluster to which each newly emerged cluster aligns. The 1000 Genomes Project populations are: YRI (Yoruba in Ibadan, Nigeria), LWK (Luhya in Webuye, Kenya), GWD (Gambian in Western Divisions in the Gambia), MSL (Mende in Sierra Leone), ESN (Esan in Nigeria), ASW (Americans of African Ancestry in SW USA), ACB (African Caribbeans in Barbados), CEU (Utah Residents with Northern and Western European Ancestry), TSI (Toscani in Italia), FIN (Finnish in Finland), GBR (British in England and Scotland), IBS (Iberian Population in Spain), GIH (Gujarati Indian from Houston, Texas), PJL (Punjabi from Lahore, Pakistan), BEB (Bengali from Bangladesh), STU (Sri Lankan Tamil from the UK), ITU (Indian Telugu from the UK), CHB (Han Chinese in Beijing, China), JPT (Japanese in Tokyo, Japan), CHS (Southern Han Chinese), CDX (Chinese Dai in Xishuangbanna, China), KHV (Kinh in Ho Chi Minh City, Vietnam), MXL (Mexican Ancestry from Los Angeles, USA), PUR (Puerto Ricans from Puerto Rico), CLM (Colombians from Medellin, Colombia), and PEL (Peruvians from Lima, Peru).

References

1. 1000 Genomes Project Consortium. A global reference for human genetic variation. Nature. 2015;526(7571):68. DOI
